# Lived experiences of psychosocial and quality of life outcomes for adults newly diagnosed with breast cancer in interior British Columbia: qualitative research

**DOI:** 10.3389/fgwh.2026.1828624

**Published:** 2026-10-21

**Authors:** Melba Sheila D’Souza, Aishwarya Manish, Hannah Nette, Juanita-Dawne R Bacsu

**Affiliations:** 1 School of Nursing and Population Health, Population Health and Aging Rural Research Centre, Thompson Rivers University, Kamloops, BC, Canada; 2 School of Population and Public Health, The University of British Columbia, Vancouver, BC, Canada; 3 Clinical Services, InspireHealth Supportive Cancer Care, Vancouver, BC, Canada; 4 School of Nursing, Canada Research Chair Tier II in Nursing and Population Health, Population Health and Aging Rural Research Centre, Thompson Rivers University, Kamloops, BC, Canada

**Keywords:** adults, breast cancer, early survivorship, oncology, psychosocial health, qualitative research, quality of life, supportive care

## Abstract

**Background:**

Breast cancer remains the most frequently diagnosed cancer worldwide, with rising incidence rates in British Columbia (BC), Canada. While survival rates have improved, the psychosocial burden of survivorship is significant. The study aimed to examine the lived experiences of psychosocial outcomes and quality of life of adults newly diagnosed with breast cancer in interior British Columbia.

**Methods:**

An explorative-descriptive qualitative research design was used. A purposive sampling was used to recruit participants who had received a diagnosis of breast cancer. Interviews were conducted with 28 participants from April 2023 to April 2024. Data were transcribed and analyzed using directed qualitative content analysis in NVivo 14 software.

**Results:**

Content analysis revealed three main categories: a) transitional care: need for mentorship, connection and advocacy; b) quality of life: psychological, emotional, and financial distress; and c) interpersonal relationships: body identity, sexuality, and dysmorphia

**Conclusions:**

As the population continues to age, insights into psychological outcomes and challenges experienced by newly diagnosed adults with breast cancer are increasingly necessary for evidence-informed knowledge and care recommendations. Healthcare professionals adopting a shared team-based, equity-oriented care approach helps to address social, financial, and physiological needs.

## 1 Introduction

Breast cancer is the most frequently diagnosed cancer globally (1) with approximately 2.3 million new diagnoses and 685,000 deaths reported in 2020 (1, 2). In British Columbia (BC), Canada, cancer accounts for 24% of annual deaths (3, 4). While advances in medical science have improved the five-year relative survival rate from 60.6% to 66.1% over the past two decades (3, 5), the transition from diagnosis to survivorship remains fraught with challenges. A breast cancer diagnosis, its treatment, and related side effects impacts the quality of life of adults (6). Adults diagnosed with breast cancer have to navigate the healthcare system while experiencing psychological stress, financial burdens, and health consequences (6). Adults with a new breast cancer diagnosis must navigate complex healthcare systems while managing psychological stress, financial toxicity, and physical side effects (7). This process is complicated by complex care coordination required to maintain the quality and continuity of care in new diagnosis (8). Hence a breast cancer diagnosis impacts quality of life (QOL) across multiple domains in adults.

Breast cancer symptoms impair one's social, occupational, and physical activities, and require practical and emotional support from family, friends, and their communities (5). Adults newly diagnosed with breast cancer often struggle with emotional expression, yet they also report experiencing emotional distress, including anxiety and depression (9). Adults newly diagnosed with breast cancer who reported receiving higher levels of emotional support also tended to report significantly lower levels of fatigue, pain interference, and depressive symptoms (7). Fisher et al. found indirect effects linking higher levels of emotional support to lower levels of depressive symptoms with lower levels of fatigue and pain interference (7). King and colleagues highlighted search for normalcy, relief from pain, fatigue, sexual issues, and sleeping difficulties, which hinder their quality of life in adults with a new breast cancer diagnosis (5). Inadequate information sharing among patients and healthcare providers (10), as well as a lack of cultural appropriate care (11) have been identified as areas for improvement.

Diagnosis of breast cancer causes fear of cancer recurrence, which triggers cognitive rumination, functional impairment, and psychological distress (12). Fear of cancer recurrence (FCR), defined as the pervasive worry or preoccupation that the malignancy will return or progress is a major psychosocial concern for adults newly diagnosed with breast cancer (12). FCR affects up to 73% of people diagnosed with breast cancer and is documented as one of the most unmet supportive care needs across the survivorship trajectory (12). Recent systematic evidence emphasizes that adults implement cognitive and behavioral mechanisms to mitigate this distress such as social and informational support-seeking, lifestyle modification, and resilience-building, alongside avoidant strategies such as thought suppression, emotional detachment, and concealing concerns from family members (12). When unaddressed, elevated FCR precipitates emotional distress, heightened healthcare utilization, and declines in health-related quality of life (13).

Breast cancer treatment often precipitates acute disruptions in self-identity and physical intimacy. In younger and reproductive-aged adults with breast cancer, body alterations, premature ovarian insufficiency, and loss of physical sensation undermine sexual health and feminine identity (14). These intimacy challenges extend beyond the diagnosis; the physiological and psychological fallout alters relational dynamics, marital communication, and caregiver well-being (15). Systemic interventions such as modified radical mastectomy, lumpectomy, chemotherapy-induced early menopause, and endocrine therapies such as aromatase inhibitors and tamoxifen induce physical alterations that alter self-concept and interpersonal intimacy (15). The loss or disfigurement of breast tissue threatens perceptions of femininity, bodily integrity, and aesthetic self-worth (14, 15). People newly diagnosed with breast cancer have diverse needs, such as information related to their health, integrated care, access to counseling or support groups, and continuity of care.

Explorations among adults newly diagnosed with breast cancer at reproductive and mid-life stages demonstrate that treatment sequelae frequently trigger an unfulfilled sexual life, marked by severe dyspareunia, vaginal dryness, marked reductions in libido, and sexual anhedonia (14). These physical and psychological traumas do not occur in isolation; breast cancer functions as a relational disease (15). Partners and spouses have illustrated that couples often experience a suspension of sexual life, wherein unfulfilled expectations, visual scars, fear of hurting the partner, and an absence of clinician-led sexual health education generate reciprocal marital strain and communication breakdowns (15). Post-treatment sexual health and body dysmorphia remain stigmatized and overlooked in oncology consultations (14, 15). These psychosocial and psychosexual vulnerabilities are compounded by geographic isolation and available oncological services.

Previous studies focused on younger demographics, and diverse impacts on workforce participation, family life, and early survivorship (16). A gap remains in understanding the differences in accommodations, information sharing and culturally appropriate care intersect with the psychosocial trauma of a new breast cancer diagnosis. The aim of the study was to examine the lived experiences of psychosocial outcomes and quality of life for adults newly diagnosed with breast cancer in interior British Columbia.

## 2 Materials and methods

### 2.1 Study design

An explorative-descriptive qualitative research design was used to provide context and facilitate a deeper understanding of the research having narrative overtones (17). The study was guided by the Standards for Reporting Qualitative Research (18). Sample Size

Sample size determination was guided by the concept of information power (19) and established benchmarks for meaning and code saturation in qualitative health research (20). Given the specific aim, homogenous adults newly diagnosed with breast cancer living in interior British Columbia, established supportive care framework (21), and dialogues elicited during 60–75 minutes semi-structured interviews, a sample of 25-28 participants was estimated to hold substantial information power.

### 2.2 Eligibility criteria

Participants considered eligible for inclusion (a) were at least 18 years old, (b) had been histologically confirmed diagnosis with primary breast cancer (Stage I, II and III) in the first year, (c) were actively undergoing or had completed primary treatment such as surgery, radiation, and chemotherapy within preceding 12 months, and (d) resided within interior British Columbia. Participants were excluded if they had self-reported distant metastasis (Stage IV), were receiving end-of-life care or palliative, or exhibited cognitive or neurological impairments that precluded informed consent and participation.

### 2.3 Ethical considerations

The BC Cancer, Interior Health and Thompson Rivers University Research Ethics Board approved this study in accordance to Tri-Council Policy Statement: Ethical Conduct for Research Involving Humans (TCPS 2) and Declaration of Helsinki. Each participant was provided with a detailed letter explaining the study's purpose and associated risks and benefits. Informed written and verbal consent was then obtained from each participant. All electronic research data were encrypted and stored on secure, password-protected servers compliant with data governance policies. Physical data were digitized and stored securely, with access restricted to the research team.

### 2.4 Data measurements

A Breast Cancer Navigation Interview (BCNI) guide was developed, validated, and structured through a three-stage process. The guide was informed by the supportive care framework (21), synthesized from recent qualitative literature on breast cancer diagnosis and survivorship. The guide was reviewed by a multidisciplinary panel consisting of two oncology nurse specialists, one behavioral psychologists, and two lived experience patients with new diagnosis of breast cancer. A pilot test with five eligible participants confirmed question clarity and emotional safety, with minor refinements made to pacing. The interview questions were arranged in a funnel progression with six items such as opening with open-ended initial diagnosis and early experience, advancing through healthcare navigation and service access, psychosocial and daily living impacts, exploring interpersonal relationships and social support, financial toxicity and occupational strain, and concluding with coping mechanisms and care recommendations. The interview questions followed a logical chronological and content progression:

a) Can you describe your experience when you were first diagnosed with breast cancer and how your care was initiated?
b) What facilitators and barriers did you experience while navigating cancer services and appointments?
c) How has your diagnosis and treatment affected your daily routine, emotional well-being, body image, and mental health?
d) In what ways has your breast cancer diagnosis influenced your relationships with family, partners, and your community?
e) What financial and occupational challenges, including travel and out-of-pocket costs, have you encountered?
f) Reflecting on your journey, what supportive resources helped you cope, and what changes would you recommend to improve care for future care?

Sociodemographic characteristics were age, gender, race/ethnicity, education, employment, marital status, years living in Canada, and living residence, and cancer treatment.

### 2.5 Data collection

Participants were recruited from Kamloops, Revelstoke, Merritt, 100 Mile, Kelowna, Vernon, Penticton and Okanagan in British Columbia through a poster announcement, community newsletter, and social media. Participants who met the inclusion criteria were recruited for the study. Twenty eight participants were screened for eligibility using purposive sampling. Interested participants were instructed to contact the trained bilingual research assistants for further information. Research assistants informed the participants about the purpose of the study and obtained written informed consent from the participants. Semi-structured interviews, which averaged about 60–75 minutes in duration, were conducted face to face and virtually between April 2023 to April 2024.

Data collection and concurrent preliminary coding proceeded until code and meaning saturation were reached. No new conceptual codes or properties were emerging regarding core challenges such as travel distress, financial toxicity, altered sexuality, and peer support deficits were found by the twenty third interview. So five additional interviews were conducted to ensure subcategory richness and disconfirming case analysis, confirming code saturation at twenty eight.

Once the data was collected, the audio files were uploaded to a secure server, with a backup copy saved to a password-protected external hard drive. Audio files were transcribed verbatim with the audio data conversion feature, then deleted from the secure server. Reflexive notes and journaling was done to record thoughts, ideas, and feelings. Data was transcribed, listened to and checked for quality audio recordings. While listening to the audio file and reading the interview transcriptions verbatim, accuracy was ensured.

### 2.6 Data analysis

NVivo 14 software was used to organize, manage, upload, store and code the data (https://www.qsrinternational.com/nvivo-qualitative-data-analysis-software/home). Data analysis was guided by Directed Qualitative Content Analysis (DQICA) to ensure the reliability, transparency, and thoroughness of the content analysis (22). A coding template was developed and used as the organizing framework for exploring the data collected and analysis.

A six-step qualitative content analytic approach and deductive-inductive framework (22, 23), guided the data analysis process to yield categories representing descriptive groupings of manifest and latent data (Figure 1). The steps included are: (1) developing a formative categorization matrix: a matrix was developed with categories and definitions formulated from supportive care need domains while permitting emergent, inductive subcategories to capture context-specific needs. To assign meaning units from the interview transcriptions, in-text identifiers were created and used, which consist of three numbers and two letters separated by a slash sign to assign meaning units in the interview transcriptions. (2) Developing a coding template: a coding template was developed to align the research objective, interview questions, and proposed data analysis. A guiding tool was used to address questions in the interview guide. (3) Specifying the unit of analysis: unit of analysis was verbatim interview transcriptions generated from audio-recorded semi-structured interviews with participants. Meaning were selected from the transcriptions where a meaning unit was considered as words, sentences, paragraphs or chunks of a text. (4) Immersion in the data: to gain an inclusive understanding of the interview transcriptions, intensive reading is conducted. Researchers familiarized themselves with the data by repeatedly reading each interview transcription and listening to the corresponding audio recordings multiple times. Researchers selected text passages that were deemed rich and thick for the aim of the study and that fit into the coding template along with notes to be analyzed in the data-analytic process. (5) Data condensation and abstraction (coding process): the meaning units were condensed, abstracted, and then coded according to the coding template. The term code was defined to assign a summative, salient, essence-capturing, and/or evocative attribute for a portion of language-based or visual data. (6) Data display: Researchers identified main categories and subcategories to explore lived experiences of participants and how they impact psychosocial and quality of life.

**Figure 1:**
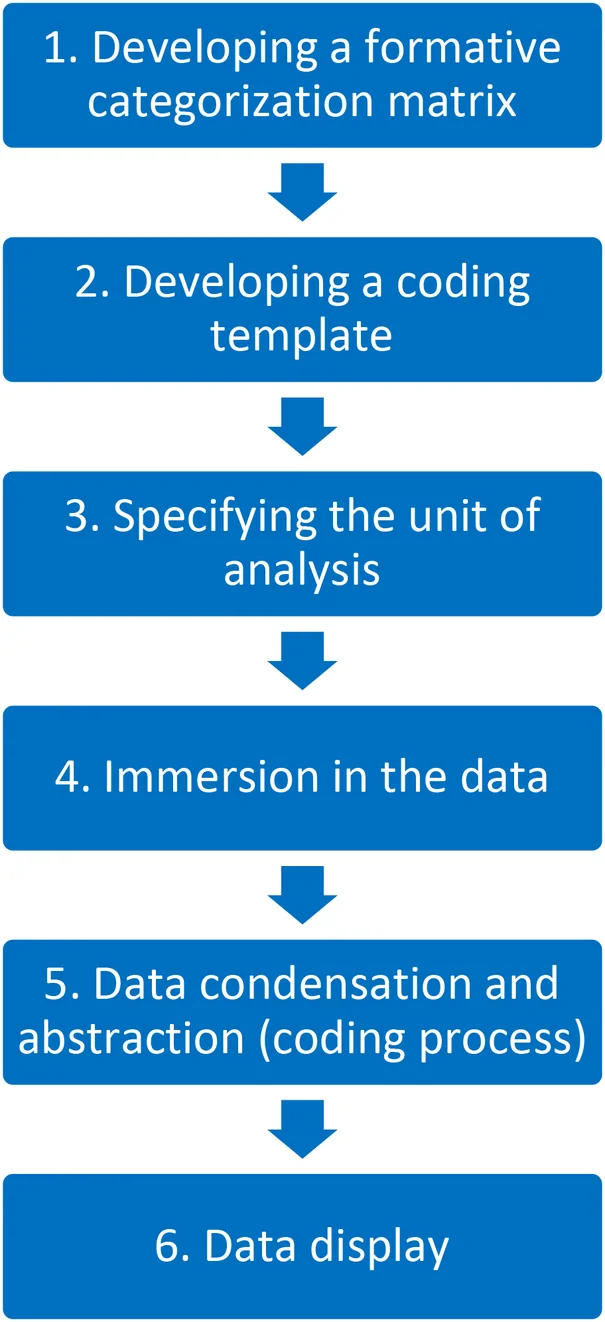
Qualitative content analytic approach and deductive-inductive framework.

### 2.7 Trustworthiness

To strengthen the trustworthiness, the Lincoln and Guba Framework was utilized throughout the research process (24). Credibility was supported by in-depth semi-structured interviews (60–75 minutes), verbatim transcription, and member checking of transcripts and synthesized content summaries were provided to all 28 participants, with 12 participants confirming interpretations and offering minor contextual refinements.

Dependability and confirmability were maintained through an audit trail logged in NVivo 14, documenting codebook development, analytic memos, and coding discrepancy reconciliations conducted by two independent coders. For transferability, participants provide detailed accounts of their perspectives and experiences verbally. If considered, the description of the data was sufficient for participants and suitable for the research objective. For authenticity, exemplary quotes were documented and included in the results, which also met this criterion.

The research team comprised of a nurse researcher, academic researcher, oncology clinician, and a research trainee with background in acute oncology and health equity in rural community. The authors recognized that their experience and familiarity with cancer treatment toxicities might predispose them to focus on clinical and physiological deficits. To mitigate this bias, reflexive journaling was maintained after each interview, and coding meetings were used to challenge interpretive assumptions and ground all categories directly in participant narratives.

## 3 Results

Twelve participants had a face-to-face interview and sixteen participants opted for an online interview to have a total of 28 participants.

### 3.1 Demographic characteristics

In the study, 32.14% of the participants were 40-49 years, 35.71% of the participants were Caucasians, and 53.57% of the participants resided in rural communities, Table 1.

**Table 1:** Sociodemographic characteristics of participants *N* = 28.

Categories	Subcategories	Frequency	Percentage
Age	18–29 years	1	3.57
	30–39 years	3	10.71
	40–49 years	9	32.14
	50–59 years	8	28.57
	60–69 years	7	25.01
Gender	Male	2	7.14
	Female	25	89.29
	2SLGBTQIA++	1	3.57
Race/ethnicity	Caucasian	10	35.71
	Indigenous	7	25
	Black	6	21.43
	Asian Minority	5	17.86
Education	High school/Diploma	11	39.29
	College/Associate/Technical	8	28.57
	Bachelor's degree	7	25
	Graduate/Maste/Doctorate degree	2	7.14
Employment	Employed	7	25
	Unemployed	6	21.43
	Retired	5	17.86
	Self-employed	2	7.14
	Social benefits	8	28.57
Marital status	Married/Common-law partner	14	50
	Divorced/Separated	6	21.43
	Widowed	6	21.43
	Single	2	7.14
Years living in Canada	Less than 10 years	8	28.57
	11–20 years	9	32.14
	More than 20 years	11	39.29
Living residence	Rural	15	53.57
	Remote	13	46.43
Cancer treatment	Surgery/radiation/chemotherapy/hormone	9	32.14
	Surgery/radiation/chemotherapy	11	39.29
	Chemotherapy/surgery/radiation/hormone	8	28.57

### 3.2 Content analysis

Three main categories and seven subcategories that emerged from the lived experiences of psychosocial outcomes and quality of life for adults newly diagnosed with breast cancer are (Figure 2):

Category 1. Transitional care: need for mentorship, connection and advocacy

- Subcategory 1. Lack of peer mentorship, lack of continuity of care, and advocacy
- Subcategory 2. Lack of professional mentorship, navigation, and support groups related to sexuality

Category 2. Quality of life: psychological, emotional, and financial distress

- Subcategory 1. Physical distress and financial burden
- Subcategory 2. Loss of work productivity and integration
- Subcategory 3. Emotional distress and fear of dying

Category 3. Interpersonal relationships: body identity, sexuality, and dysmorphia

- Subcategory 1. Body identity and body image crises
- Subcategory 2. Shift in sexuality and fear of rejection

**Figure 2:**
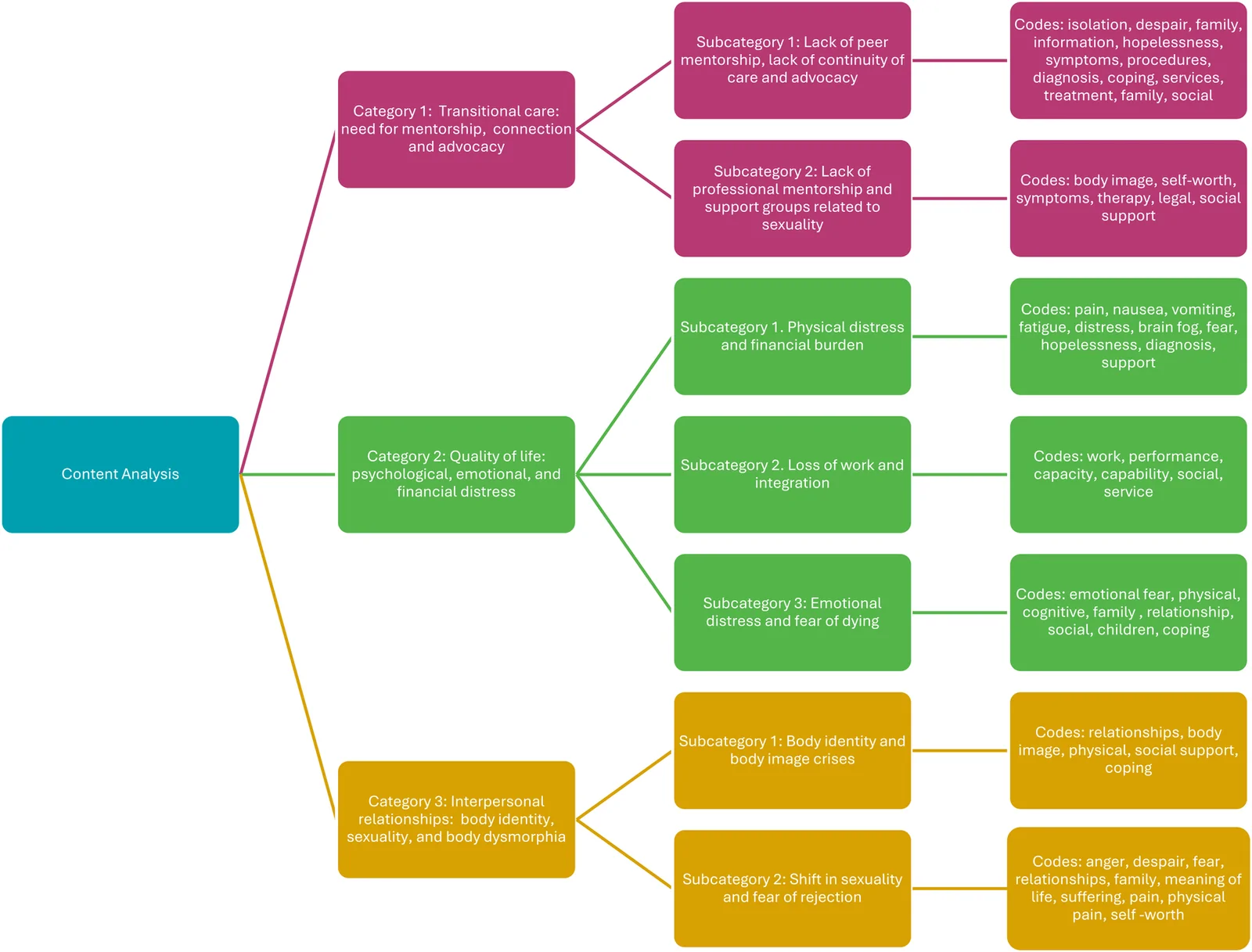
Content analysis of categories, subcategories, and codes.

#### 3.2.1 Category 1. Transitional care: need for mentorship, connection and advocacy

##### 3.2.1.1 Subcategory 1. Lack of peer mentorship, continuity of care, and advocacy

Participants identified a lack of formal mentorship, lack of continuity of care and advocacy as contributing to feelings of isolation, despair, and uncertainty which impacted their ability to cope with their new diagnoses.

“Once I finished the chemo sessions after my first breast cancer diagnosis, I asked if I was cured and was told cancer never goes away it goes into remission. The doctor I spoke to was brief and dismissive. I felt like I had been cut off and left on my own to manage my recovery. I asked my surgeon to follow up with me for 5 years. It was actually her who discovered recurrent breast cancer, a high-grade ductal carcinoma insitu 2 deposits 1.5 cm and 6 mm both left breasts, I had a bilateral mastectomy based on my family history. She was amazing and took all my questions seriously. Since I do carry the gene BRCA2 she agreed to see my daughter, aged 33. I really did not have a peer person or navigator until I joined the Spirit Warriors Breast Cancer Survivor, they have been my salvation. I was not made aware of information and programs I could have perhaps used.” P#2

This excerpt described experiences of participant feeling disconnected, abandoned, and hopeless, which highlighted lack of advocacy and accountability for new diagnosis.

“I was getting yearly mammogram procedure until the government said it was not necessary, and I only required every 2-year assessment. Had I been able to have had my last mammogram my new breast cancer diagnosis, would have been caught at an early stage. Now because of this, I was diagnosed with stage 3. I am furious that I did not have the information of coping**.** I have not had regular services after breast cancer treatments which are concerning. I feel I should be monitored especially since I take Tamoxifen which is known to cause other cancers and blood clots.” P#9

The participant expressed the need for better attention to informational coping, gaining a sense of control, and monitoring.

“My family doctor who gave me my new cancer diagnosis moved shortly afterwards leaving me with no family doctor for 2 years. Health records or files were not passed along from his practice to new General Practitioner. There is a need for more health information, support groups and patient advocacy positions, it is so hard to fight the system and services to feel heard. I suffered and fought hard because I had a 2-month-old baby, I wanted to thrive and live with managing my care. Child care, managing my home and life for single parents is important. Also joining a peer or professional support group for the kids and spouses or partners are important to learn through the stages of the new diagnosis, procedures, and effects of treatment.” P#15

Translational care, connections, integration, and advocacy are required to ensure that voice of newly diagnosed patients are heard. Participants identified the challenges of a new diagnosis with balancing work, caring for their children and change in lifestyle in the absence of family and social support. Hence, mentorship, navigation and support groups were required for adults with a new diagnosis of breast cancer.

##### 3.2.1.2 Subcategory 2. Lack of professional mentorship and support groups related to sexuality

Participants identified a distinct lack of professional led mentorship and support and increasing need for more accessible support groups to explore body image, self-worth, and sexual health in the wake of a new breast cancer diagnosis.

“Morning support group of women with new breast cancer. A program for women experiencing body dysmorphia and depression post diagnosis. and most importantly, a course on intimacy, sex and sexuality changes on aromatase inhibitors, as one's ability to have sex and intimacy completely goes away.” P#3

“Evening support groups in the hospital is the only group that meets which is far too late for those of us who tire easily. I want a group that specifically deals with breast cancer symptoms and sexuality as aromatase inhibitors greatly ruin sex lives, but no-one talks about it.” P#12

“I did not receive any therapy and counselling. I would plan for nurse navigators or mentors, emotional support counsellors, pharmacy consultations to deal with medication queries, physio and massage therapy, financial counselling, legal help, social support, and support for my family and caregivers on an as needed, ongoing basis for new breast cancer diagnosis.” P#21

Integrating professional mentorship and support groups in sexual health programs would improve the psychosocial well-being for adults with newly diagnosed breast cancer. Participants identified a need for more inclusive support groups to accommodate explorations of sexuality and intimacy.

#### 3.2.2 Category 2. Quality of Life: Psychological, Emotional, and Financial Distress

##### 3.2.2.1 Subcategory 1. Physical distress and financial burden

Participants described heightened feelings of self-consciousness and sensitivity related to the perceived loss of femininity and sexuality, as well as body dysmorphia, which had negatively impacted their emotional well-being.

“I am at the hospital up to three times a week and it is so stressful trying to find parking when I am so sick. It is also so expensive to keep having to pay each time I go. I would love to have a women's support group that is not at 7 pm. That is far too late for someone in active breast cancer diagnosis and treatment who tires so easily. I really feel there needs to be a program for women to help with body dysmorphia post mastectomy. I know many women whined about the late hours. I would be happy to volunteer to organize such a program. Lastly, there was no-one to speak to regarding how to use Cannabidiol (CBD) to help with extreme pain, nausea, vomiting, fatigue, and distress post chemotherapy.” P#23

This excerpt describes the financial pressure the participants experienced in relation to caregivers taking paid or unpaid leave from work to provide care and support. Financial toxicity was related to lost wages and depleted savings.

“I had sick days at work and then partial sick term disability after my new breast cancer diagnosis, so I did not lose my job. I definitely had less income, especially when my husband took unpaid leave from work and early retirement to be here for me. It was monetarily tight and difficult going back to part time work hours due to ongoing fear of recurrence, fatigue and brain fog. As happened at the latter part of my career, I had sick days banked, and most big bills paid such as mortgages and university education for my two kids.” P#14

Participants also expressed their own fear, hopelessness and frustrations with returning to work, citing ongoing fatigue, malaise, active treatment, and the fear of recurrence associated with limiting their capacity and ability to work and to ensure their job security, financial stability and safety net.

“My breast cancer diagnosis has led to loss of work productivity and income during my first diagnosis and treatment, and I had to avail myself of unpaid work and then retire early on receiving an aggressive breast cancer diagnosis, leading to huge loss of income, loss of pension funding, relying on savings and RRSP's much sooner than expected. Breast cancer has depleted a lot of my RRSPs. I have had to pay out-of-pocket for many supportive care services, transportation, and accommodation while receiving diagnostics, treatment and care in other locations. My life insurance costs have increased dramatically overnight. I cannot qualify for travel insurance any longer.” P#17

Integrating into normal work was difficult due to the treatment distress, fatigue, and lifestyle changes. Participants associated their breast cancer diagnosis with loss of income, sickness/disability benefits, early retirement, and the depletion of financial savings. The non-eligibility for employer insurance and government programs without benefits lead to financial distress. Out-of-pocket expenses related to illness exacerbated financial sources leading to increased mortgage and debt.

##### 3.2.2.2 Subcategory 2. Loss of work productivity and integration

Participants described obtaining extended period of time away from work and traveling long distances to larger centres for diagnostic appointments, medical care and treatment plans leading to financial and psychological strain.

“I had to take time off for surgery after my new breast cancer diagnosis. I was unable to meet my performance and unable to work full-time during my treatment which resulted in wage losses, which then meant that we had to borrow money on our line of credit to pay for household expenses and repairs. I also had to make multiple trips to XXX for appointments, which took time and money, and then stayed at the Cancer Lodge while undergoing radiation, which was another enormous expense.” P#1

“The aggressiveness of my new breast cancer and the length of my treatment and surgeries prevented me from working to my full capacity, returning to my capabilities and continuing my career. I had to take intended time off work leading to loss of pay while pursuing diagnostics, oncologist visits, radiation treatment effects, and seeking healthcare.” P#19

Frequent follow-up visits and access to specialized cancer care required extended periods away from home and work creating anxiety and depression in addition to financial strain.

“I have had to take longer periods of being away from work and seek social disability benefit and assistance to continue my healthcare, seek ongoing care for my new breast cancer. My mother came and lived with me for 2 months while I recovered from a double mastectomy. She looked after my son as my husband had to work. When she was not available my husband had to take days off work.” P#6

Financial and economic constraints were associated with inability to return to usual work and loss of work days following a new diagnosis, coping with treatment effects, and use healthcare services.

##### 3.2.2.3 Subcategory 3. Emotional distress and fear of dying

Participants described experiencing fear of recurrence and fear of dying leading to emotional distress. Participants discussed the extent to which treatment distress limited their physical and cognitive functioning such as brain fog, which impacted spouses/partners and family members who faced work disruption, marital relationships and reduced family activities. The side effects of treatment served as reminders of limiting family events, socialization and recreation.

“My children fear contacting breast cancer, and I fear my own mortality. Complacency is gone. Fear of recurrence affects my state of mind, sense of control and quality of life. Dealing with new diagnosis, treatment effects and distress impairs my brain functioning, mobility, ability to travel and ability to spend long hours with grandkids. I am less able to do the things I had planned in my retirement because of ongoing costs for supportive care. Monthly cancer clinic visits and cycles of chemotherapy limit my ability to make safety net and security plans for family, socialization and travel.” P#7

“Aggressive chemotherapy and radiation treatments, multiple surgeries, infections, treatment side effects to heart and lungs have affected my overall health and quality of life. The treatments after the new breast cancer diagnosis saved my life; however, the short- and long-term side effects have caused enduring distress and substantial negative effects on me and my quality of life.” P# 25

Financial changes disrupted retirement plans, which had economic impacts exacerbated by fear of the unknown and stressors associated with fear of recurrence. The maladaptive coping mechanism of fear of death caused rumination. There was a need for increased psychosocial support, financial assistance, and flexible treatment scheduling for better emotional satisfaction and quality of life.

#### 3.2.3 Category 3. Interpersonal relationships: body identity, sexuality, and dysmorphia

##### 3.2.3.1 Subcategory 1. Body identity and body image crises

Interpersonal relationship such as perceived loss of identity and lack of independence was connected to the challenges of engaging in meaningful activities. The affection and gratitude displayed highlighted the importance of strong kinship and emotional support in coping with new diagnoses. The physical alterations of cancer treatment resulted in identity crises, compounded by a lack of privacy, and need for psychotherapy and counseling.

“The new breast cancer has impacted me in my ways of thinking, knowing and doing. I am no longer able to do my favorite things, like cooking, gardening, playing sports and going on vacations, because of treatment distress. The effect of the treatment is also hard on my kids, family and friends, who have to take extra cautions and care of me while I am sick and recovering from side effects and preventing complications. My life has been altered by breast cancer, but I am still grateful for the support of my loved ones as we work through this difficult time together.” P#11

“At the moment of diagnosis, I felt uneasy about the future. The diagnosis and treatment process were physically exhausting, especially the side effects of chemotherapy and radiation. However, as the treatment began, it cost me physical and emotional changes that I needed to adapt to. I joined a breast cancer support groups to help me cope with the lymphedema, hair loss, losing my breasts, menopause, sexual discomfort, and lack of attractiveness created anxiety and isolation. I had follow-ups to check for signs of recurrence. Breast cancer treatment prompted me to rethink my life goals and priorities. I gradually found a positive outlook on life.” P5

Participants experienced a loss of self-esteem and self-doubt leading to isolation and withdrawal. Participants reported feeling of emotional ambivalence and a shift from fighting to acceptance and independence to dependency, leading to increased locus of control. The tension to keep a brave and calm face with family members resulted in emotional suppression and expressions of gratitude. Strong spousal/partner, caregiver, and social support networks improved emotional well-being.

“The first thing that was impacted by new breast cancer was my view of myself as a person and as a human. I used to be very independent, assertive and confident in my abilities, but now I fear failure, loss of control, impostor syndrome and am constantly second-guessing myself. In the beginning, it was difficult to explain to my loved ones and people how I was feeling, what I was going through; so, most of my friends and family were left in the dark about how sick I really was and what the diagnosis meant to me. I did not want them to sympathize, feel confused and uncomfortable around me, so I tried to hide my inner emotions and keep up physical appearances as much as possible to look and feel normal which ultimately left me incomplete and impacted my life.” P#10

Excerpts showed how participant's desired to regain confidence and maintain normalcy following acceptance and realization of the new diagnosis had significantly impacted them. Participants expressed difficulty communicating their needs, which led to emotional alienation and isolation. This change in attitude and increased vulnerability highlighted the need for reaffirmation and social networking. A new cancer diagnosis often created barriers to transparent and honest communication, which impacted family and social relationships in the absence of expressive emotional coping.

##### 3.2.3.2 Subcategory 2. Shift in sexuality and fear of rejection

Changes in sexuality and surgical scars contributed to decreased confidence, leading to unexplained feelings of anger, despair, fear a withdrawal from intimacy. Participants described difficulty adapting and adjusting to unexpected changes in their bodies, financial situations, and interpersonal relationships.

“Breast cancer diagnosis will always affect my family, and meaning of life. It makes changes in how I do things, how I think of things and how I behave and act to reduce suffering and pain. There are always bodily changes such as sexuality and fear of intimacy. I lost a year of work and used savings which has made living more difficult. It is hard to feel confident with permanent body scars, so I do not necessarily chase the idea of exploring intimacy and sexual relationships.” P#13

“I have experienced fluctuations sometimes for better or for worse. The fear and uncertainty during my new diagnosis created anxiety and depression throughout treatment. I have been struggling with hearing voices, and I would have liked to be more aware how to identify and manage this better. I developed greater resilience and learned to cope with the changing emotions. The communication with the doctor and nurse helped me to participate in decisions, understand treatment and side-effects, and express my own needs and to care for myself.” P#8

“A new cancer diagnosis and its treatment have severely affected my life in many ways. Physically, psychologically, mentally, emotionally have all been the result of breast cancer. My memory has been quite affected, and my physical confidence has diminished. Losing my hair and sexuality was extremely traumatic and the surgeries and permanent scars I bear are a daily reminder of my very recent and new diagnosis. I felt ashamed and embarrassed when I had no hair and lacked femininity. I still keep my breasts covered so my husband does not see the way my breasts look as I had nipple reconstruction that is not symmetrical, and I have no areolas. I get instantly emotionally distressed when I think and talk about my cancer journey as I have known anxiety, depression and post-traumatic stress disorder. When I have unexplained anger, aches, pains and mood changes, I think that the cancer has returned and I live in fear that it will come back one day to haunt me, gone are the days of looking and feeling good about myself.” P#16

The financial burden from lost income added to the emotional strain such as low self-worth and negative body image, and participants described experiencing a shift in their perceived locus of control and sense of well-being due in difficulties with emotional regulation and financial struggles.

Participants reported psychological distress in the wake of their breast cancer diagnoses surrounding hypervigilance, self-worth, body image, trauma, and fear of recurrence. Hair loss, loss of sexuality, and surgical scars led to feelings of shame and embarrassment, which exacerbated poor self-esteem and the inability to maintain sexual relationships. Hair loss and surgical scars were tangible reminders of treatment distress, and difficulty regaining assertiveness and confidence.

## 4 Discussion

There was a demand for morning support groups, managing work fatigue and increasing engagement in care that addressed body dysmorphia, fatigue and sexuality immediately post-mastectomy. The findings identified how the lack of concealment and the cost of frequent travel to urban settings creating emotional and financial distress, initiated early retirement and the exhaustion of financial savings.

### 4.1 Category 1. Transitional care: need for mentorship, connection and advocacy

Mentorship programs have been found to help people find new purposes and make sense of their experiences, which transforms their uncertainty into something constructive and affirming (16). The need for increasing supportive care is closely related to mentorship (25). Study findings highlighted a strong desire for guidance among breast cancer survivors during times of uncertainty. Participants indicated a need for guidance from professional navigators whose job is to support them in accessing resources and making them aware of programs that support informed decision-making as patients navigate new diagnoses. This finding aligned with existing research that emphasized the importance of patient navigation to address deficits in breast cancer survivorship (26, 27). Professional navigation is an important component of improved access to care, reported outcomes, and quality of life (28). The positive impact of participation in support group fostered a sense of belonging and well-being.

Participants described finding valued when well-positioned and connected with peers with new breast cancer diagnosis. Alfieri and colleagues discussed the need for peer group support for newly diagnosed breast cancer who sought comfort and compassion talking to peers with lived experiences (29). Our study findings showed that participants required flexible meeting times, especially for morning support groups, to accommodate the fatigue associated with cancer treatment and to meet immediate needs. The participant highlighted the importance of advocacy, autonomy and self-determination. Patient advocates supported healthy communication and relationship between patients and physicians, which improved trust, decision-making, and seeking health information within rural communities (30).

For the participants, continuous care and ongoing support were important from the point of diagnosis through every stage of treatment, including reconstruction, rehabilitation, and early survivorship. Other studies showed that being diagnosed with breast cancer for the first time and being younger than 40 years were predictors of higher complementary therapy (31). Continuity of care was associated with a decrease in supportive care needs and a higher quality of life in adults newly diagnosed with breast cancer (32). Continuity of care emerged as an important part of reducing the impact of treatment distress for newly diagnosed patients.

The value of peer mentorship and professional navigation reflected a need to mitigate the isolation and abandonment experienced after diagnosis and treatment completion. Participants expressed feeling abruptly disconnected from the healthcare service following active treatment, a transition frequently documented as a vulnerable and uncomfortable period when newly diagnosed (4, 28). Formal and informal peer connections, such as dragon boating and support groups, provided essential emotional validation and communal normalization. Seeking social and informational support served as a coping strategy for navigating post-treatment uncertainty and fear of cancer recurrence (12). Connecting with peers who experienced distress alleviated feeling of isolation and helped to connect ambiguous physical symptoms without immediately resorting to catastrophic interpretations (12, 33).

### 4.2 Category 2. Quality of life: psychological, emotional, and financial distress

Participants experienced fatigue as a barrier to achieving psychosocial health and engaging in meaningful activities. Out-of-pocket expenses and loss of income created financial strain and impacted quality of life. Other studies demonstrated that age, education, time of diagnosis, and symptoms were significantly associated with better psychosocial support (31). The financial strain due to out-of-pocket expenses such as parking, travel, food, and lodging necessities was a major source of psychological stress. Taking time off work and having to traveling long distances for medical appointments, diagnostics, and treatment was a source of financial stress for people with newly diagnosed breast cancer. These perceptions are consistent with studies demonstrating financial toxicity and diminished quality of life of adults diagnosed with breast cancer (31).

Specialized surgical, diagnostic, and radiation therapies necessitated traveling longer distances exceeding to tertiary cancer centers in rural communities. The cumulative expenses incurred with fuel, food, and accommodation, combined with unpaid work leave or involuntary early retirement, generated acute financial toxicity (25, 34). These burdens intensified psychological distress, sleep disturbances, and existential fears of recurrence and fear of dying. Mardani et al. (12) identified that managing the threat of cancer recurrence required extensive lifestyle and occupational adaptations. Our study findings showed participants reported reduced work hours, altered household responsibilities, and withdrew from work due to fatigue and fear of compromising physical recovery as demonstrated in other studies (12). Our findings of longer travel distances requiring extended periods away from familial support and work environments, caused financial strain shifting from logistical inconvenience to psychological stressors that impeded emotional recovery as suggested by other studies (12, 25).

Evidence demonstrated that interprofessional oncology teams integrated medical oncologists, nurse navigators, oncology nurses, psycho-oncologists, and oncology social workers reduced fragmentation, decreased unnecessary travel, and lowered distress scores (26, 28). Randomized and observational evaluations of decentralized nurse-led navigation and telehealth community oncology networks have shown enhanced symptom control, improved adherence to therapies, and reductions in out-of-pocket travel expenditures (25, 27). Implementing such interprofessional networks is essential to deliver equitable supportive care that shielded adults newly diagnosed with breast cancer from financial toxicity.

### 4.3 Category 3. Interpersonal relationships: body identity, sexuality, and dysmorphia

Participants reported experiencing self-consciousness and sensitivity related to body dysmorphia and perceived changes in their femininity and sexuality, which negatively impacted their emotional well-being. Our study findings align with other studies that treatment significantly affected body image and identity (35). Miniotti et al. identified a need to address self-image and to manage the changes associated with a breast cancer diagnosis such as shifts in body image, intimacy, and sexuality due to dysfunction and a loss of interpersonal desire (2). Healthcare professionals must assess readiness for education and commitment to change for care recommendations based on specific needs and preferences (6). While some of our participants described difficulty communicating their sexual experiences as the cause of their emotional isolation and alienation, other participants engaged in maladaptive coping techniques such as withdrawal and isolation, which impacted their ability to strengthen spousal/partner and family connections. Our study described participants perception of suppressing grief and projecting an outward façade of resilience to protect family members from emotional burden. Our findings reflect repressive coping styles and emotional suppression strategies, wherein participants minimized conscious distress to preserve interpersonal harmony and family stability during life-threatening illness (36)

Our findings demonstrated how treatment and surgical scarring sexual self-concept and interpersonal connection. These findings illustrate how altered body integrity precipitated sexual dysfunction and avoidance behaviors as suggested in other studies (14). This treatment distress extended dyadically as demonstrated in other studies that partners/spouses experienced confusion, grief, and communicative paralysis regarding sexual changes, illustrating the necessity for couples-based sexual health supportive care (15). The intrusive worry over fear of recurrence identified in our finding underlined that unmanaged fear fueled psychological illness in the absence of targeted cognitive-behavioral and peer navigation coping strategies (12).

Participants in our study described psychological distress resulted from alopecia, surgical scarring, asymmetric breast reconstruction, and therapy side effects, which eroded bodily integrity and self-confidence. Our findings showed that bodily disruption impaired sexual functioning and intimacy for participants who identified mastectomies and suppressive therapies that lead to unfulfilled sexual life as demonstrated by other studies (14) characterized by dyspareunia, marked loss of erogenous breast sensation, vaginal atrophy, and feelings of lost femininity. Other studies showed that adults with new breast cancer diagnosis engaged in avoidance of intimate contact and experienced embarrassment when undressed before their partners (14).

Our findings showed that participants were reluctant to express their sexual and physical needs to their partners, which reflected the relational complexity (15). When spouses experienced changes in physical intimacy, a lack of clear professional communication resulted in a mutual sexual life suspension, marked by unfulfilled expectations, partner hesitation stemming from fear of causing physical pain, and suppression of desire (15). Other studies showed that healthcare providers rarely initiated conversations regarding post-treatment sexual rehabilitation, premature menopause, and safe intimacy aids (14). This physician-patient silence reinforced the taboo surrounding post-cancer sexuality, leaving participants to manage altered embodiment and marital strain in their own ways of knowing and being (15).

Some participants in our study described masking their internal distress and displaying a brave face to protect children, partners, and community members from emotional distress. This masked behaviour illustrated the contextual enactment of avoidant and repressive coping mechanisms (12, 37). Avoidant coping strategies such as hiding concerns, emotional withdrawal, and suppressing intrusive cancer thoughts were deployed to seek emotional equilibrium and relational harmony (12). While emotional concealment preserved family functioning and shield loved ones from distress, prolonged use of avoidance prevented authentic emotional processing and hindered access to seeking professional help and psychosocial support (12, 34).

### 4.4 Limitations

The study recruited participants from interior British Columbia; while reflective of rural and remote communities, the findings may not fully translate to urban communities with proximity to specialized cancer centers. Purposive sampling through community posters and survivor networks introduced self-selection and volunteer bias, favored participants who possessed health literacy and support networks, while potentially omitted participants who experienced psychosocial isolation and cancer distress. The cross-sectional qualitative design limited insights into supportive care needs across breast cancer continuum. Majority of the participants were female; the psychosocial and intimacy challenges of male breast cancer remain unaddressed. Cultural and gender diversity was limited, non-binary individuals were not underrepresented, and ethnocultural differences in coping and sexual discourse need further research investigation.

### 4.5 Recommendations

Oncology programs must integrate professional nurse mentorship and navigation from the point of diagnostic biopsy to assist adults newly diagnosed with breast cancer with care coordination, survivorship resource, and anticipatory guidance. Healthcare teams should routinely utilize standardized emotional, psychosocial distress and financial toxicity screening. Cancer supportive care should implement specialized survivorship counseling to address treatment distress from cardiac, endocrine, menopause, reproductive, body image, and sexual health. Professional support groups should be structured with accessible and inclusive options and flexible scheduling to accommodate treatment-induced fatigue, cognitive exhaustion, family and work commitments.

Provincial health authorities should expand financial protection programs and travel lodging subsidies such as travel grants and bursaries for newly diagnosed adults with breast cancer requiring tertiary cancer care. Healthcare delivery services must prioritize shared team-based, equity-oriented care models that establish decentralized hybrid community oncology care networks, combining virtual specialized consultations with local community clinics and oncology nurses to reduce the travel burden and health disparities experienced by adults newly diagnosed with breast cancer.

Further investigations should adopt longitudinal prospective designs to evaluate how supportive care for newly diagnosed adults with breast cancer needs evolve across the survivorship continuum. A mixed-methods studies is needed to evaluate the cost-effectiveness and psychosocial efficacy of professional navigation, digital health and digital therapeutics tailored for adults newly diagnosed with breast cancer in rural and remote communities.

## 5 Conclusion

Adults newly diagnosed with breast cancer were impacted by physiological distress, emotional distress, and financial distress living in rural and remote communities. Physical and psychosocial outcomes are linked to access and use of healthcare and community services leading to better quality of life. These findings highlight capacity building, professional mentorship, and transitional care to improve connection and advocacy, interpersonal relationships, and quality of life. The study emphasized the value of communication and relationships highlighting the importance of supportive care tailored to the needs and preferences of adults with newly diagnosed with breast cancer in rural and remote communities.

## Data Availability

The original contributions presented in the study are included in the article/supplementary material, further inquiries can be directed to the corresponding author.
